# Bromido(quinolin-8-ol-κ^2^ *N*,*O*)(quinolin-8-olato-κ^2^ *N*,*O*)zinc(II) methanol monosolvate

**DOI:** 10.1107/S160053681003672X

**Published:** 2010-09-18

**Authors:** Ezzatollah Najafi, Mostafa M. Amini, Seik Weng Ng

**Affiliations:** a Department of Chemistry, General Campus, Shahid Beheshti University, Tehran 1983963113, Iran; b Department of Chemistry, University of Malaya, 50603 Kuala Lumpur, Malaysia

## Abstract

The title compound, [ZnBr(C_9_H_6_NO)(C_9_H_7_NO)]·CH_3_OH, has its metal atom *N*,*O*-chelated by a neutral and a deproton­ated 8-hy­droxy­quinoline ligand. The hy­droxy unit of the neutral ligand is a hydrogen-bond donor to the methanol O atom and the alk­oxy O atom of the monoanionic ligand is a hydrogen-bond acceptor to the methanol O atom. In the crystal, adjacent mol­ecules are linked by these two hydrogen bonds, generating a chain running along the *a* axis.

## Related literature

For a related structure, see: Najafi *et al.* (2010 ▶).
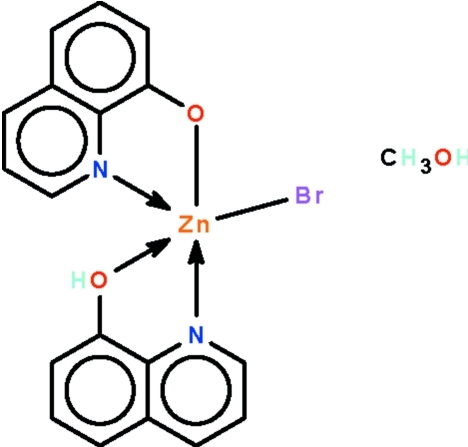


## Experimental

### 

#### Crystal data

[ZnBr(C_9_H_6_NO)(C_9_H_7_NO)]·CH_4_O
*M* *_r_* = 466.63
Triclinic, 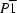

*a* = 8.4485 (7) Å
*b* = 8.6968 (7) Å
*c* = 13.1868 (10) Å
α = 97.241 (1)°
β = 99.209 (1)°
γ = 109.470 (1)°
*V* = 884.81 (12) Å^3^
*Z* = 2
Mo *K*α radiation
μ = 3.67 mm^−1^
*T* = 100 K
0.30 × 0.20 × 0.10 mm

#### Data collection

Bruker SMART APEX diffractometer
Absorption correction: multi-scan (*SADABS*; Sheldrick, 1996 ▶) *T*_min_ = 0.406, *T*_max_ = 0.711
8392 measured reflections
4022 independent reflections
3354 reflections with *I* > 2σ(*I*)
*R*_int_ = 0.028

#### Refinement

*R*[*F* ^2^ > 2σ(*F* ^2^)] = 0.032
*wR*(*F* ^2^) = 0.103
*S* = 1.10
4022 reflections
237 parameters
H-atom parameters constrained
Δρ_max_ = 0.78 e Å^−3^
Δρ_min_ = −0.85 e Å^−3^

### 

Data collection: *APEX2* (Bruker, 2009 ▶); cell refinement: *SAINT* (Bruker, 2009 ▶); data reduction: *SAINT*; program(s) used to solve structure: *SHELXS97* (Sheldrick, 2008 ▶); program(s) used to refine structure: *SHELXL97* (Sheldrick, 2008 ▶); molecular graphics: *X-SEED* (Barbour, 2001 ▶); software used to prepare material for publication: *publCIF* (Westrip, 2010 ▶).

## Supplementary Material

Crystal structure: contains datablocks global, I. DOI: 10.1107/S160053681003672X/bt5356sup1.cif

Structure factors: contains datablocks I. DOI: 10.1107/S160053681003672X/bt5356Isup2.hkl

Additional supplementary materials:  crystallographic information; 3D view; checkCIF report

## Figures and Tables

**Table 1:** Hydrogen-bond geometry (Å, °)

*D*—H⋯*A*	*D*—H	H⋯*A*	*D*⋯*A*	*D*—H⋯*A*
O2—H2⋯O3^i^	0.84	1.90	2.585 (4)	137
O3—H3⋯O1	0.84	1.71	2.551 (4)	178

Symmetry code: (i) 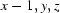.

**Fig. 1.**
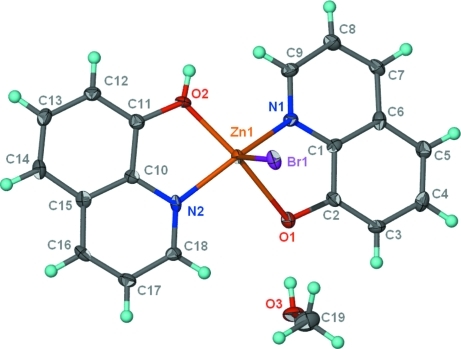
Anisotropic displacement ellipsoid plot (Barbour, 2001) of the title compound at the 70% probability level. Hydrogen atoms are drawn as spheres of arbitrary radius.

**Fig. 2.**
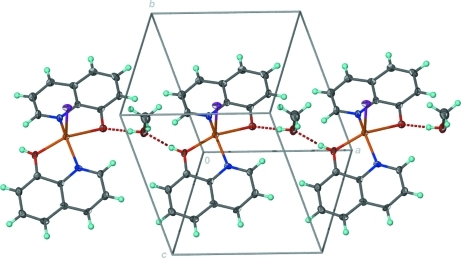
Hydrogen bonded chain structure.

## supplementary crystallographic information

### Comment

An earlier study reported C_10_H_10_NO^+.^ZnBr_2_(C_10_H_8_NO)^.^CH_3_OH, which feature cations, tetrahedral anions and solvent molecules linked by N···O, O···O and O···Br hydrogen bonds into a linear chain. The salt was synthesized by reacting zinc bromide and 2-methyl-8-hydroxyquinoline in methanol; no base was added (Najafi *et al.*, 2010). The present study uses 8-hydoxyquinoline instead of 2-methy-8-hydroxyquinoline as the organic reactant. The product is a mono-solavated neutral molecule (Scheme I, Fig. 1). The methanol-solvated compound, ZnBr(C_9_H_6_NO)(C_9_H_7_NO)^.^CH_3_OH, has its metal atom *N*,*O*-chelated by a neutral and deprotonated 8-hydroxyquinoline ligand. The hydroxy unit of the neutral ligand is hydrogen-bond donor methanol O atom and the alkoxy O atom of the monoanionic ligand is hydrogen-bond acceptor to methanol O atom. Adjacent molecules are linked by these two hydrogen bonds to generate a linear chain running along the *a*-axis of the triclinic unit cell (Fig. 2).

### Experimental

Zinc bromide (0.19 g, 0.75 mmol) and 8-hydroxyquinoline (0.22 g, 1.5 mmol) were loaded into a convection tube; the tube was filled with dry methanol and kept at 333 K. Crystals were collected from the side arm after several days.

### Refinement

Hydrogen atoms were placed in calculated positions (C–H 0.95–0.98, O–H 0.84 Å) and were included in the refinement in the riding model approximation, with *U*(H) set to 1.2–1.5*U*_eq_(C,*O*).

### Crystal data

**Table:**

[ZnBr(C_9_H_6_NO)(C_9_H_7_NO)]·CH_4_O	*Z* = 2
*M**_r_* = 466.63	*F*(000) = 468
Triclinic, *P*1	*D*_x_ = 1.751 Mg m^−^^3^
Hall symbol: -P 1	Mo *K*α radiation, λ = 0.71073 Å
*a* = 8.4485 (7) Å	Cell parameters from 3867 reflections
*b* = 8.6968 (7) Å	θ = 2.5–28.2°
*c* = 13.1868 (10) Å	µ = 3.67 mm^−^^1^
α = 97.241 (1)°	*T* = 100 K
β = 99.209 (1)°	Prism, yellow
γ = 109.470 (1)°	0.30 × 0.20 × 0.10 mm
*V* = 884.81 (12) Å^3^	

### Data collection

**Table:**

Bruker SMART APEX diffractometer	4022 independent reflections
Radiation source: fine-focus sealed tube	3354 reflections with *I* > 2σ(*I*)
graphite	*R*_int_ = 0.028
ω scans	θ_max_ = 27.5°, θ_min_ = 2.5°
Absorption correction: multi-scan (*SADABS*; Sheldrick, 1996)	*h* = −10→10
*T*_min_ = 0.406, *T*_max_ = 0.711	*k* = −11→11
8392 measured reflections	*l* = −15→17

### Refinement

**Table:**

Refinement on *F*^2^	Primary atom site location: structure-invariant direct methods
Least-squares matrix: full	Secondary atom site location: difference Fourier map
*R*[*F*^2^ > 2σ(*F*^2^)] = 0.032	Hydrogen site location: inferred from neighbouring sites
*wR*(*F*^2^) = 0.103	H-atom parameters constrained
*S* = 1.10	*w* = 1/[σ^2^(*F*_o_^2^) + (0.0457*P*)^2^ + 1.9422*P*] where *P* = (*F*_o_^2^ + 2*F*_c_^2^)/3
4022 reflections	(Δ/σ)_max_ = 0.001
237 parameters	Δρ_max_ = 0.78 e Å^−^^3^
0 restraints	Δρ_min_ = −0.85 e Å^−^^3^

### Fractional atomic coordinates and isotropic or equivalent isotropic displacement parameters (Å^2^)

**Table:**

	*x*	*y*	*z*	*U*_iso_*/*U*_eq_	
Zn1	0.45338 (5)	0.64466 (5)	0.71737 (3)	0.01184 (12)	
Br1	0.58054 (4)	0.92415 (4)	0.81357 (3)	0.01654 (11)	
O1	0.6864 (3)	0.6465 (3)	0.64187 (19)	0.0152 (5)	
O2	0.2092 (3)	0.5306 (3)	0.73396 (19)	0.0145 (5)	
H2	0.1230	0.5461	0.7024	0.022*	
O3	0.9908 (3)	0.6800 (3)	0.7299 (2)	0.0209 (6)	
H3	0.8908	0.6707	0.7016	0.031*	
N1	0.3868 (4)	0.6687 (4)	0.5653 (2)	0.0119 (6)	
N2	0.5025 (4)	0.4869 (3)	0.8093 (2)	0.0121 (6)	
C1	0.5180 (4)	0.7280 (4)	0.5148 (3)	0.0110 (6)	
C2	0.6796 (4)	0.7212 (4)	0.5573 (3)	0.0128 (7)	
C3	0.8163 (4)	0.7842 (4)	0.5120 (3)	0.0150 (7)	
H3A	0.9255	0.7819	0.5415	0.018*	
C4	0.7946 (5)	0.8531 (5)	0.4209 (3)	0.0177 (7)	
H4	0.8897	0.8950	0.3892	0.021*	
C5	0.6384 (4)	0.8603 (4)	0.3777 (3)	0.0151 (7)	
H5	0.6264	0.9080	0.3172	0.018*	
C6	0.4957 (5)	0.7963 (4)	0.4238 (3)	0.0141 (7)	
C7	0.3290 (5)	0.7915 (4)	0.3821 (3)	0.0153 (7)	
H7	0.3085	0.8350	0.3208	0.018*	
C8	0.1969 (5)	0.7238 (5)	0.4304 (3)	0.0173 (7)	
H8	0.0835	0.7166	0.4020	0.021*	
C9	0.2328 (4)	0.6652 (4)	0.5232 (3)	0.0156 (7)	
H9	0.1412	0.6208	0.5571	0.019*	
C10	0.3596 (4)	0.4025 (4)	0.8429 (3)	0.0109 (6)	
C11	0.2035 (4)	0.4283 (4)	0.8012 (3)	0.0138 (7)	
C12	0.0569 (4)	0.3427 (4)	0.8339 (3)	0.0150 (7)	
H12	−0.0481	0.3560	0.8074	0.018*	
C13	0.0599 (5)	0.2365 (5)	0.9055 (3)	0.0165 (7)	
H13	−0.0433	0.1800	0.9263	0.020*	
C14	0.2083 (5)	0.2119 (4)	0.9464 (3)	0.0165 (7)	
H14	0.2079	0.1400	0.9951	0.020*	
C15	0.3606 (4)	0.2956 (4)	0.9146 (3)	0.0128 (7)	
C16	0.5206 (5)	0.2803 (4)	0.9519 (3)	0.0145 (7)	
H16	0.5282	0.2085	0.9996	0.017*	
C17	0.6636 (5)	0.3682 (4)	0.9194 (3)	0.0153 (7)	
H17	0.7716	0.3604	0.9457	0.018*	
C18	0.6497 (4)	0.4706 (4)	0.8467 (3)	0.0134 (7)	
H18	0.7494	0.5301	0.8235	0.016*	
C19	1.0704 (5)	0.8335 (5)	0.8036 (3)	0.0271 (9)	
H19A	1.0933	0.8107	0.8743	0.041*	
H19B	0.9936	0.8967	0.8004	0.041*	
H19C	1.1789	0.8984	0.7867	0.041*	

### Atomic displacement parameters (Å^2^)

**Table:**

	*U*^11^	*U*^22^	*U*^33^	*U*^12^	*U*^13^	*U*^23^
Zn1	0.0117 (2)	0.0145 (2)	0.0111 (2)	0.00508 (15)	0.00377 (15)	0.00635 (15)
Br1	0.0208 (2)	0.01368 (17)	0.01481 (19)	0.00510 (14)	0.00314 (14)	0.00568 (13)
O1	0.0162 (12)	0.0189 (12)	0.0138 (12)	0.0078 (10)	0.0056 (10)	0.0078 (10)
O2	0.0105 (11)	0.0194 (12)	0.0163 (13)	0.0080 (10)	0.0012 (9)	0.0076 (10)
O3	0.0130 (12)	0.0228 (13)	0.0256 (15)	0.0072 (11)	−0.0002 (11)	0.0035 (11)
N1	0.0106 (13)	0.0158 (14)	0.0101 (14)	0.0054 (11)	0.0026 (11)	0.0030 (11)
N2	0.0159 (14)	0.0129 (13)	0.0080 (13)	0.0037 (11)	0.0045 (11)	0.0049 (11)
C1	0.0104 (15)	0.0115 (15)	0.0109 (16)	0.0041 (12)	0.0033 (12)	−0.0001 (12)
C2	0.0159 (17)	0.0138 (15)	0.0117 (16)	0.0083 (13)	0.0044 (13)	0.0042 (13)
C3	0.0115 (16)	0.0187 (17)	0.0171 (18)	0.0080 (13)	0.0034 (13)	0.0043 (14)
C4	0.0172 (18)	0.0193 (17)	0.0155 (18)	0.0026 (14)	0.0076 (14)	0.0052 (14)
C5	0.0169 (17)	0.0161 (16)	0.0107 (16)	0.0044 (14)	0.0015 (13)	0.0030 (13)
C6	0.0185 (17)	0.0126 (15)	0.0114 (16)	0.0046 (13)	0.0061 (14)	0.0027 (13)
C7	0.0180 (17)	0.0180 (17)	0.0099 (16)	0.0072 (14)	−0.0003 (13)	0.0043 (13)
C8	0.0159 (17)	0.0236 (18)	0.0126 (17)	0.0076 (14)	0.0010 (14)	0.0061 (14)
C9	0.0120 (16)	0.0209 (17)	0.0161 (18)	0.0077 (14)	0.0045 (14)	0.0045 (14)
C10	0.0124 (15)	0.0120 (15)	0.0089 (15)	0.0056 (12)	0.0025 (12)	0.0011 (12)
C11	0.0145 (16)	0.0158 (16)	0.0118 (16)	0.0062 (13)	0.0025 (13)	0.0034 (13)
C12	0.0108 (16)	0.0208 (17)	0.0123 (17)	0.0054 (13)	0.0003 (13)	0.0038 (13)
C13	0.0146 (17)	0.0199 (17)	0.0152 (18)	0.0041 (14)	0.0063 (14)	0.0062 (14)
C14	0.0206 (18)	0.0153 (16)	0.0152 (18)	0.0056 (14)	0.0069 (14)	0.0066 (14)
C15	0.0151 (16)	0.0123 (15)	0.0082 (16)	0.0029 (13)	0.0004 (13)	−0.0002 (12)
C16	0.0197 (18)	0.0145 (16)	0.0127 (17)	0.0105 (14)	0.0021 (14)	0.0048 (13)
C17	0.0143 (17)	0.0208 (17)	0.0127 (17)	0.0093 (14)	0.0009 (13)	0.0043 (14)
C18	0.0121 (16)	0.0171 (16)	0.0143 (17)	0.0081 (13)	0.0049 (13)	0.0045 (13)
C19	0.0193 (19)	0.027 (2)	0.029 (2)	0.0051 (16)	0.0007 (17)	−0.0014 (17)

### Geometric parameters (Å, °)

**Table:**

Zn1—O2	2.030 (2)	C7—C8	1.372 (5)
Zn1—N2	2.040 (3)	C7—H7	0.9500
Zn1—N1	2.050 (3)	C8—C9	1.411 (5)
Zn1—O1	2.340 (2)	C8—H8	0.9500
Zn1—Br1	2.3911 (5)	C9—H9	0.9500
O1—C2	1.362 (4)	C10—C15	1.409 (5)
O2—C11	1.328 (4)	C10—C11	1.445 (5)
O2—H2	0.8400	C11—C12	1.383 (5)
O3—C19	1.430 (5)	C12—C13	1.404 (5)
O3—H3	0.8400	C12—H12	0.9500
N1—C9	1.319 (4)	C13—C14	1.378 (5)
N1—C1	1.372 (4)	C13—H13	0.9500
N2—C18	1.322 (4)	C14—C15	1.410 (5)
N2—C10	1.361 (4)	C14—H14	0.9500
C1—C2	1.414 (5)	C15—C16	1.417 (5)
C1—C6	1.418 (5)	C16—C17	1.364 (5)
C2—C3	1.368 (5)	C16—H16	0.9500
C3—C4	1.422 (5)	C17—C18	1.406 (5)
C3—H3A	0.9500	C17—H17	0.9500
C4—C5	1.376 (5)	C18—H18	0.9500
C4—H4	0.9500	C19—H19A	0.9800
C5—C6	1.416 (5)	C19—H19B	0.9800
C5—H5	0.9500	C19—H19C	0.9800
C6—C7	1.412 (5)		
			
O2—Zn1—N2	82.51 (11)	C6—C7—H7	120.1
O2—Zn1—N1	95.88 (11)	C7—C8—C9	118.8 (3)
N2—Zn1—N1	143.75 (11)	C7—C8—H8	120.6
O2—Zn1—O1	150.98 (10)	C9—C8—H8	120.6
N2—Zn1—O1	90.06 (10)	N1—C9—C8	123.4 (3)
N1—Zn1—O1	73.91 (10)	N1—C9—H9	118.3
O2—Zn1—Br1	112.41 (7)	C8—C9—H9	118.3
N2—Zn1—Br1	109.66 (8)	N2—C10—C15	122.9 (3)
N1—Zn1—Br1	104.40 (8)	N2—C10—C11	116.5 (3)
O1—Zn1—Br1	96.52 (6)	C15—C10—C11	120.7 (3)
C2—O1—Zn1	108.4 (2)	O2—C11—C12	124.4 (3)
C11—O2—Zn1	111.3 (2)	O2—C11—C10	118.4 (3)
C11—O2—H2	124.4	C12—C11—C10	117.2 (3)
Zn1—O2—H2	124.4	C11—C12—C13	121.4 (3)
C19—O3—H3	109.5	C11—C12—H12	119.3
C9—N1—C1	118.4 (3)	C13—C12—H12	119.3
C9—N1—Zn1	122.8 (2)	C14—C13—C12	121.9 (3)
C1—N1—Zn1	117.2 (2)	C14—C13—H13	119.0
C18—N2—C10	119.0 (3)	C12—C13—H13	119.0
C18—N2—Zn1	129.8 (2)	C13—C14—C15	118.6 (3)
C10—N2—Zn1	110.8 (2)	C13—C14—H14	120.7
N1—C1—C2	117.6 (3)	C15—C14—H14	120.7
N1—C1—C6	122.0 (3)	C10—C15—C14	120.1 (3)
C2—C1—C6	120.3 (3)	C10—C15—C16	116.4 (3)
O1—C2—C3	124.0 (3)	C14—C15—C16	123.5 (3)
O1—C2—C1	116.0 (3)	C17—C16—C15	120.2 (3)
C3—C2—C1	119.9 (3)	C17—C16—H16	119.9
C2—C3—C4	119.9 (3)	C15—C16—H16	119.9
C2—C3—H3A	120.0	C16—C17—C18	119.3 (3)
C4—C3—H3A	120.0	C16—C17—H17	120.4
C5—C4—C3	121.2 (3)	C18—C17—H17	120.4
C5—C4—H4	119.4	N2—C18—C17	122.2 (3)
C3—C4—H4	119.4	N2—C18—H18	118.9
C4—C5—C6	119.7 (3)	C17—C18—H18	118.9
C4—C5—H5	120.2	O3—C19—H19A	109.5
C6—C5—H5	120.2	O3—C19—H19B	109.5
C7—C6—C5	123.5 (3)	H19A—C19—H19B	109.5
C7—C6—C1	117.5 (3)	O3—C19—H19C	109.5
C5—C6—C1	118.9 (3)	H19A—C19—H19C	109.5
C8—C7—C6	119.8 (3)	H19B—C19—H19C	109.5
C8—C7—H7	120.1		
			
O2—Zn1—O1—C2	94.6 (3)	C4—C5—C6—C7	−177.0 (3)
N2—Zn1—O1—C2	169.1 (2)	C4—C5—C6—C1	0.9 (5)
N1—Zn1—O1—C2	22.0 (2)	N1—C1—C6—C7	−4.1 (5)
Br1—Zn1—O1—C2	−81.1 (2)	C2—C1—C6—C7	176.7 (3)
N2—Zn1—O2—C11	6.3 (2)	N1—C1—C6—C5	177.9 (3)
N1—Zn1—O2—C11	149.9 (2)	C2—C1—C6—C5	−1.3 (5)
O1—Zn1—O2—C11	82.7 (3)	C5—C6—C7—C8	178.5 (3)
Br1—Zn1—O2—C11	−101.9 (2)	C1—C6—C7—C8	0.6 (5)
O2—Zn1—N1—C9	21.6 (3)	C6—C7—C8—C9	2.0 (5)
N2—Zn1—N1—C9	106.9 (3)	C1—N1—C9—C8	−1.9 (5)
O1—Zn1—N1—C9	173.8 (3)	Zn1—N1—C9—C8	162.7 (3)
Br1—Zn1—N1—C9	−93.4 (3)	C7—C8—C9—N1	−1.4 (6)
O2—Zn1—N1—C1	−173.6 (2)	C18—N2—C10—C15	−1.2 (5)
N2—Zn1—N1—C1	−88.3 (3)	Zn1—N2—C10—C15	−174.5 (3)
O1—Zn1—N1—C1	−21.3 (2)	C18—N2—C10—C11	178.6 (3)
Br1—Zn1—N1—C1	71.4 (2)	Zn1—N2—C10—C11	5.2 (4)
O2—Zn1—N2—C18	−178.6 (3)	Zn1—O2—C11—C12	175.2 (3)
N1—Zn1—N2—C18	91.6 (3)	Zn1—O2—C11—C10	−5.4 (4)
O1—Zn1—N2—C18	29.5 (3)	N2—C10—C11—O2	0.1 (5)
Br1—Zn1—N2—C18	−67.5 (3)	C15—C10—C11—O2	179.8 (3)
O2—Zn1—N2—C10	−6.2 (2)	N2—C10—C11—C12	179.6 (3)
N1—Zn1—N2—C10	−96.0 (3)	C15—C10—C11—C12	−0.7 (5)
O1—Zn1—N2—C10	−158.1 (2)	O2—C11—C12—C13	−179.9 (3)
Br1—Zn1—N2—C10	104.9 (2)	C10—C11—C12—C13	0.7 (5)
C9—N1—C1—C2	−176.1 (3)	C11—C12—C13—C14	−0.2 (6)
Zn1—N1—C1—C2	18.4 (4)	C12—C13—C14—C15	−0.3 (5)
C9—N1—C1—C6	4.7 (5)	N2—C10—C15—C14	179.9 (3)
Zn1—N1—C1—C6	−160.8 (3)	C11—C10—C15—C14	0.2 (5)
Zn1—O1—C2—C3	161.4 (3)	N2—C10—C15—C16	0.5 (5)
Zn1—O1—C2—C1	−20.0 (3)	C11—C10—C15—C16	−179.2 (3)
N1—C1—C2—O1	3.7 (4)	C13—C14—C15—C10	0.3 (5)
C6—C1—C2—O1	−177.1 (3)	C13—C14—C15—C16	179.7 (3)
N1—C1—C2—C3	−177.7 (3)	C10—C15—C16—C17	1.0 (5)
C6—C1—C2—C3	1.6 (5)	C14—C15—C16—C17	−178.5 (3)
O1—C2—C3—C4	177.1 (3)	C15—C16—C17—C18	−1.7 (5)
C1—C2—C3—C4	−1.4 (5)	C10—N2—C18—C17	0.4 (5)
C2—C3—C4—C5	1.0 (6)	Zn1—N2—C18—C17	172.3 (3)
C3—C4—C5—C6	−0.8 (5)	C16—C17—C18—N2	1.0 (5)

### Hydrogen-bond geometry (Å, °)

**Table:**

*D*—H···*A*	*D*—H	H···*A*	*D*···*A*	*D*—H···*A*
O2—H2···O3^i^	0.84	1.90	2.585 (4)	137
O3—H3···O1	0.84	1.71	2.551 (4)	178

Symmetry codes: (i) *x*−1, *y*, *z*.
